# Supplementary material for: A Humanized Yeast Phenomic Model of Deoxycytidine Kinase to Predict Genetic Buffering of Nucleoside Analog Cytotoxicity
Source: Genes (Basel). 2019 Sep 30;10(10):770. doi: 10.3390/genes10100770 (PMC6826991; doi:10.3390/genes10100770)
Supplement: Supplementary file 1 [file genes-10-00770-s001.zip › Additional_Files/Additional_File7_GOTermHeatmaps.zip!/Additional_File7_GOTermHeatmaps/single-stranded_DNA_binding.pdf]

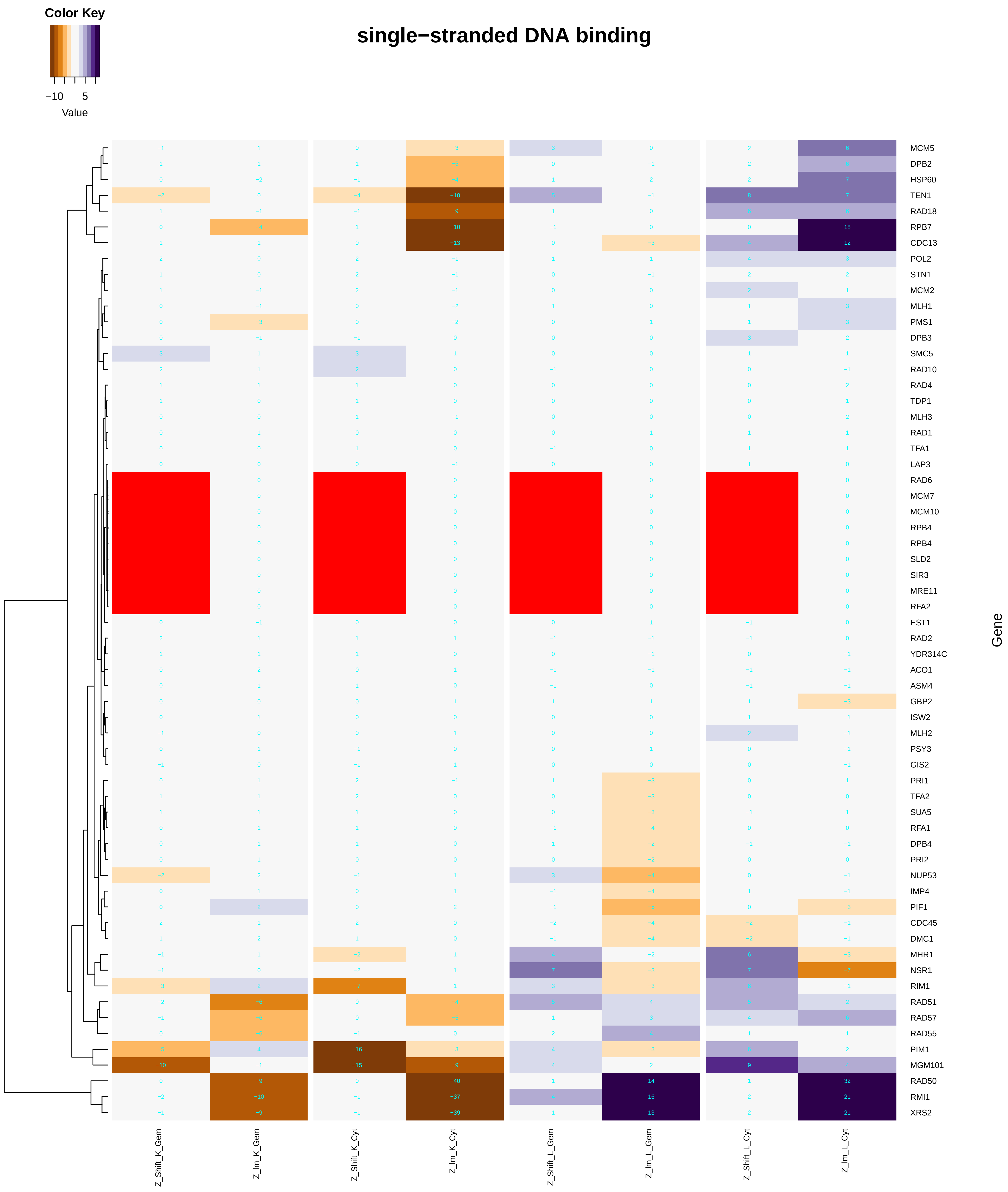
single−stranded DNA binding
Color Key
−10
5
Value
MCM5
DPB2
HSP60
TEN1
RAD18
RPB7
CDC13
POL2
STN1
MCM2
MLH1
PMS1
DPB3
SMC5
RAD10
RAD4
TDP1
MLH3
RAD1
TFA1
LAP3
RAD6
MCM7
MCM10
RPB4
RPB4
SLD2
SIR3
MRE11
RFA2
EST1
RAD2
YDR314C
ACO1
ASM4
GBP2
ISW2
MLH2
PSY3
GIS2
PRI1
TFA2
SUA5
RFA1
DPB4
PRI2
NUP53
IMP4
PIF1
CDC45
DMC1
MHR1
NSR1
RIM1
RAD51
RAD57
RAD55
PIM1
MGM101
RAD50
RMI1
XRS2
Gene
Z_Shift_K_Gem
Z_Im_K_Gem
Z_Shift_K_Cyt
Z_Im_K_Cyt
Z_Shift_L_Gem
Z_Im_L_Gem
Z_Shift_L_Cyt
Z_Im_L_Cyt

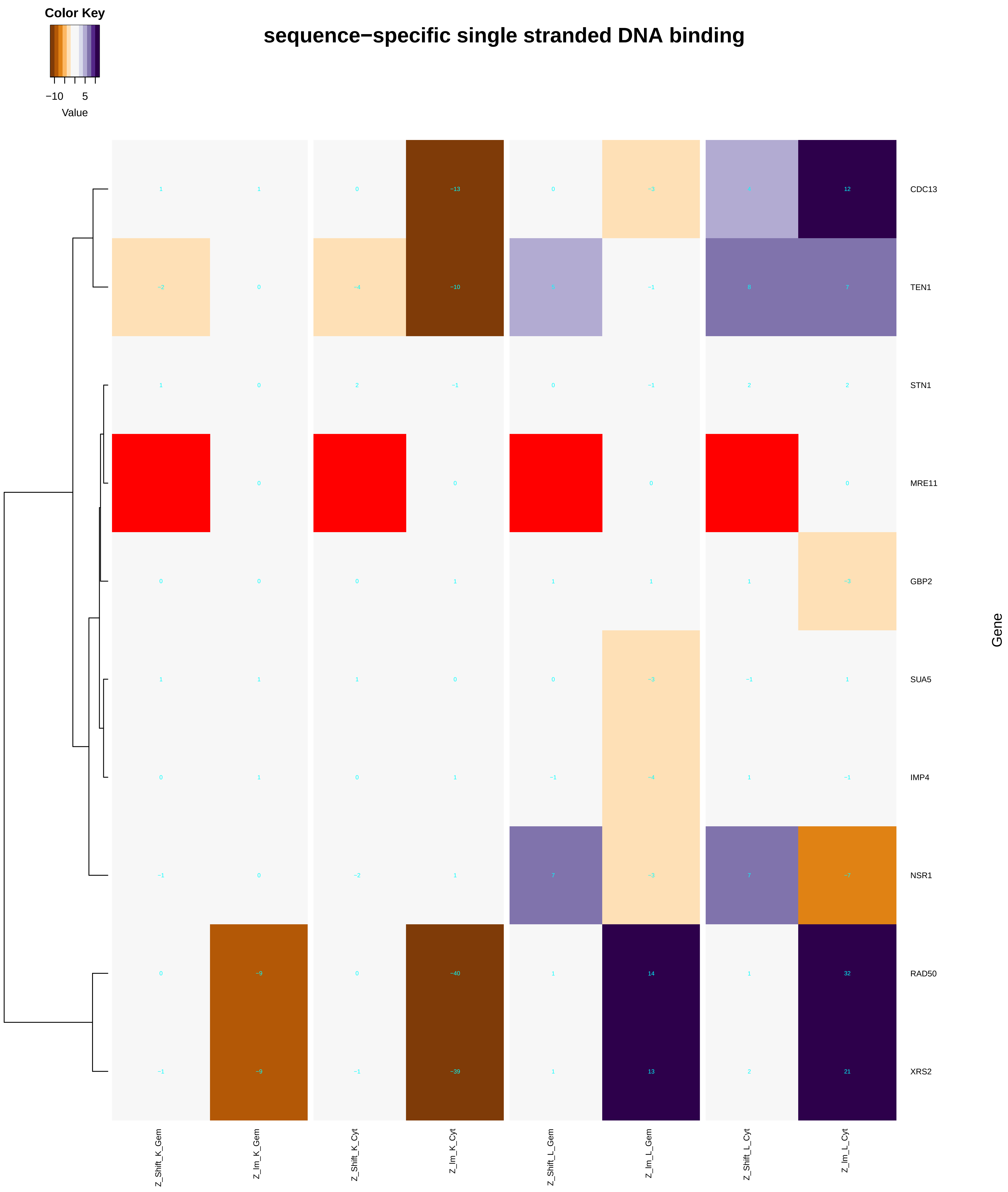

Color Key
−10
5
Value
sequence−specific single stranded DNA binding
CDC13 1 1 0 −13 0 −3 12
TEN1 −2 0 −4 −10 −1 8 7
STN1 1 0 2 −1 0 −1 2 2
MRE11 0 0 0 0
GBP2 0 0 0 1 1 1 1 −3
SUA5 1 1 1 0 0 −3 −1 1
IMP4 0 1 0 1 −1 −4 1 −1
NSR1 −1 0 −2 1 7 −3 7 −7
RAD50 0 −9 0 −40 1 14 1 32
XRS2 −1 −9 −1 −39 1 13 2 21
Gene
Z_Shift_K_Gem
Z_Im_K_Gem
Z_Shift_K_Cyt
Z_Im_K_Cyt
Z_Shift_L_Gem
Z_Im_L_Gem
Z_Shift_L_Cyt
Z_Im_L_Cyt

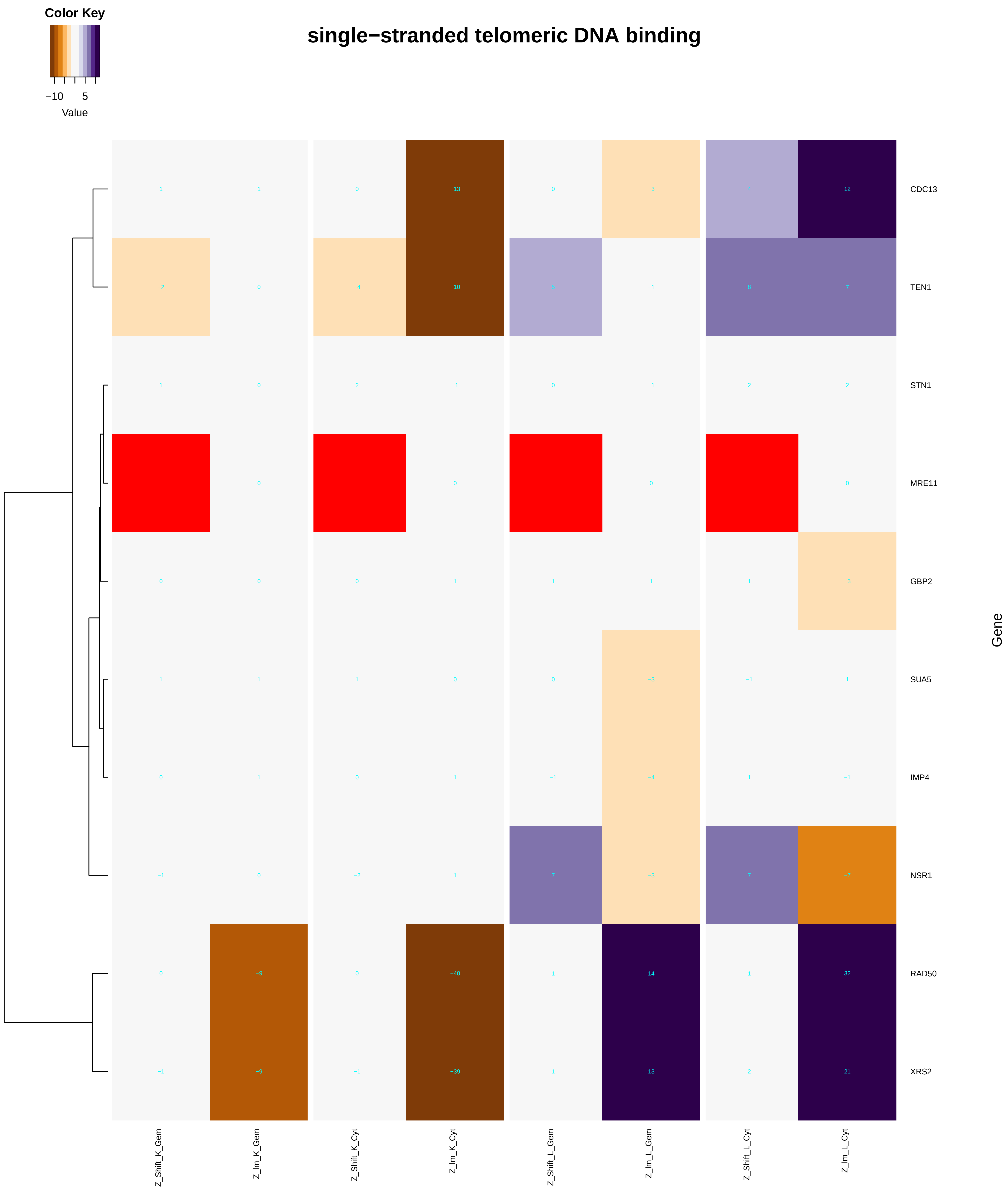
Color Key
−10
5
Value
single−stranded telomeric DNA binding
CDC13
TEN1
STN1
MRE11
GBP2
SUA5
IMP4
NSR1
RAD50
XRS2
Gene
Z_Shift_K_Gem
Z_Im_K_Gem
Z_Shift_K_Cyt
Z_Im_K_Cyt
Z_Shift_L_Gem
Z_Im_L_Gem
Z_Shift_L_Cyt
Z_Im_L_Cyt
